# A Physiologically Based Model of Orexinergic Stabilization of Sleep and Wake

**DOI:** 10.1371/journal.pone.0091982

**Published:** 2014-03-20

**Authors:** Ben D. Fulcher, Andrew J. K. Phillips, Svetlana Postnova, Peter A. Robinson

**Affiliations:** 1 School of Physics, The University of Sydney, Sydney, New South Wales, Australia; 2 Division of Sleep Medicine, Brigham and Women’s Hospital, Harvard Medical School, Boston, Massachusetts, United States of America; 3 Center for Integrated Research and Understanding of Sleep, The University of Sydney, Sydney, New South Wales, Australia; 4 Brain Dynamics Center, The University of Sydney, Sydney, New South Wales, Australia; 5 Cooperative Research Center for Alertness, Safety and Productivity, The University of Sydney, Sydney, New South Wales, Australia; Simon Fraser University, Canada

## Abstract

The orexinergic neurons of the lateral hypothalamus (Orx) are essential for regulating sleep-wake dynamics, and their loss causes narcolepsy, a disorder characterized by severe instability of sleep and wake states. However, the mechanisms through which Orx stabilize sleep and wake are not well understood. In this work, an explanation of the stabilizing effects of Orx is presented using a quantitative model of important physiological connections between Orx and the sleep-wake switch. In addition to Orx and the sleep-wake switch, which is composed of mutually inhibitory wake-active monoaminergic neurons in brainstem and hypothalamus (MA) and the sleep-active ventrolateral preoptic neurons of the hypothalamus (VLPO), the model also includes the circadian and homeostatic sleep drives. It is shown that Orx stabilizes prolonged waking episodes via its excitatory input to MA and by relaying a circadian input to MA, thus sustaining MA firing activity during the circadian day. During sleep, both Orx and MA are inhibited by the VLPO, and the subsequent reduction in Orx input to the MA indirectly stabilizes sustained sleep episodes. Simulating a loss of Orx, the model produces dynamics resembling narcolepsy, including frequent transitions between states, reduced waking arousal levels, and a normal daily amount of total sleep. The model predicts a change in sleep timing with differences in orexin levels, with higher orexin levels delaying the normal sleep episode, suggesting that individual differences in Orx signaling may contribute to chronotype. Dynamics resembling sleep inertia also emerge from the model as a gradual sleep-to-wake transition on a timescale that varies with that of Orx dynamics. The quantitative, physiologically based model developed in this work thus provides a new explanation of how Orx stabilizes prolonged episodes of sleep and wake, and makes a range of experimentally testable predictions, including a role for Orx in chronotype and sleep inertia.

## Introduction

Since the discovery of the orexin A and orexin B neurotransmitters (also termed hypocretin 1 and 2) by Sakurai *et al.* [1] and de Lecea *et al.* [2] in 1998, the orexinergic neurons of the lateral hypothalamic area (Orx) have been implicated in a wide range of neurological processes, including a key role in the regulation of sleep and wake [3], [4]. The orexins have also been shown to have a role in feeding, emotion, reward function, and motivation [1], [5]–[8]. The neurodegenerative disorder narcolepsy [9] is characterized by a loss of approximately 90% of Orx [10], and is a condition that affects approximately 0.05% of the population [11]. It is thought that some process–perhaps an autoimmune attack [12]–selectively destroys these orexinergic cells [13]. Narcolepsy is characterized by awakenings during sleep, unintentional naps during wake, drowsiness, and difficulty in waking from sleep [14]. The condition is often accompanied by cataplexy, the sudden loss of muscle tone triggered by strong emotions [15], although over one quarter of all narcoleptics do not have cataplexy [11], perhaps due to less severe loss of Orx [16], [17].

Although the link between the loss of Orx and narcolepsy has been established and the key neurological pathways of Orx are known, the mechanisms through which loss of Orx causes narcoleptic symptoms remain unclear [18]. For example, it is commonly thought that Orx excites the wake-promoting monoaminergic neurons (MA) during wake and thereby acts to stabilize the sleep-wake switch [19], [20], but it is not clear how Orx also stabilizes sleep, the destabilization of which is a hallmark of narcolepsy [15]. Homeostatic control of sleep in narcoleptics is thought to be normal, since they exhibit normal recovery from sleep deprivation and have a normal total daily sleep duration [9]. The underlying circadian dynamics in both orexin knockout mice and narcoleptic humans also appears to be normal [9], [21]. Thus, despite apparently normal homeostatic and circadian processes, a reduction in Orx somehow produces ‘behavioral state instability’, with low thresholds to transition between sleep and wake [9], [22]. In this work, we present a detailed, physiologically justified explanation of this phenomenon and explain how the loss of Orx gives rise to these characteristically low thresholds for behavioral state transitions in narcolepsy.

Phenomenological models of sleep-wake dynamics that have built upon Borbély’s two-process model [23] have been successful in predicting a range of sleep-wake behaviors [24], including subjective fatigue during sleep deprivation, internal desynchronization, fragmented sleep during continuous bedrest, and the sleep durations of shift workers [25], [26]. However, incorporating Orx into such models is problematic because they lack a physiological framework. In contrast, physiologically based models of sleep represent the neuronal populations and their interactions explicitly, allowing new physiological information to be incorporated straightforwardly. Following advances in the understanding of key sleep-regulatory nuclei in the brainstem and hypothalamus [27], [28], a range of physiologically based sleep models have been developed [29]–[34]. In this work we build on the Phillips-Robinson model of the sleep-wake switch [35], which is based on the mutually-inhibitory sleep-active ventrolateral preoptic nucleus (VLPO) and the wake-active monoaminergic hypothalamic and brainstem neuronal populations (MA). The model produces flip-flop dynamics between sleep and wake, as driven by homeostatic and circadian processes. Although sleep/wake dynamics are known to be regulated by a variety of processes [36], the Phillips-Robinson model captures the core dynamics of the sleep-wake switch, which turns out to be a powerful approximation. Despite being fitted using a relatively small set of behavioral and physiological data, the model has predicted the results of many experiments, while providing insights into the physiological dynamics that underly its predictions, including sleep deprivation [37], [38], sleep fragmentation [39], caffeine intake [40], mammalian sleep [41], shift work [42], and internal desynchrony [43], and has successfully predicted sleep latencies [37], arousal thresholds [39], and subjective fatigue levels [38].

The paper is structured as follows. First we explain the mathematical formulation of the new model in terms of the relevant physiology. The dynamics of the model are then characterized in terms of net drives to the sleep-active VLPO and the wake-active MA: 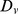 and 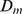, respectively. Different combinations of these drives are shown to control whether: (i) the system is awake, (ii) the system is asleep, or (iii) sleep and wake are simultaneously stable, with characteristic thresholds for transitions between the states. These results allow us to explain how Orx’s known mechanisms, including exciting the MA, relaying a circadian signal to the MA, and being inhibited by the VLPO, all act to stabilize extended bouts of sleep and wake. By including noise in the model and simulating the loss of Orx, we show that the model generates increasingly fragmented sleep-wake time series, as is characteristic of narcolepsy. Finally we show that dynamics resembling sleep inertia result from including Orx in the model, and we link the timescale of this gradual sleep-to-wake transition to that of Orx dynamics.

## Models

In this section, we develop a new sleep model that includes Orx, giving a non-mathematical overview of the physiology and model structure first, and then providing further mathematical details. The new model is an extension of an existing model by Phillips and Robinson [35], which has been characterized in detail previously [37], [39]. The model includes the interactions between three key neuronal populations: VLPO, MA, and Orx, as well as the circadian and homeostatic drives. Although a wide range of processes are thought to regulate sleep [36], VLPO, MA, and Orx are known to play central roles [19], and here we show that many salient features of healthy and pathological sleep can be captured by considering just this reduced system. Note that because we do not distinguish between REM sleep and different stages of NREM sleep (since the physiological basis for these dynamics are yet to be pinned down [44]), we do not attempt to model transitions between NREM and REM sleep stages, nor any effect of Orx on the frequency and timing of these transitions [19], [21]. The link between Orx loss and cataplexy [15] is also not investigated here; we group the monoaminergic nuclei as a uniform population, whereas cataplexy involves a discoordination of firing activity across the monoaminergic nuclei [45]. Such dynamics could be explored in future work (cf. [44]), but here we focus solely on the dynamics of sleep and wake.

### Physiology and Model Overview

The flip-flop dynamics of sleep and wake are proposed to result from the mutual inhibition of wake-active MA and sleep-active VLPO [27], [46]. The MA group includes nuclei that use monoaminergic neurotransmitters: the histaminergic tuberomammillary nucleus (TMN), norepinephrinergic locus coeruleus (LC), serotoninergic dorsal raphé nucleus (DR), and dopaminergic ventral tegmental area (VTA) [47]–[49]. Orx excites the MA during wake [8], [19], [50]. Monoaminergic neurotransmitters inhibit the VLPO, and the VLPO inhibits the MA via GABAergic projections [4], [51], [52]. Due to the mutual inhibition between the MA and VLPO populations, only a single population is active at any one time, and the dynamics resemble that of an electronic flip-flop circuit [27]. This provides the basis for consolidated bouts of either sleep (active MA, suppressed VLPO) or wake (active VLPO, suppressed MA), with the active population determined by the net inputs, or *drives*, to each population. Although populations other than the VLPO have been implicated as having a role in inducing and/or maintaining sleep, including the median preoptic nuclei (MnPO) [19], melanin-concentrating hormone cells in the hypothalamus [53], neurons in the striatum and globus pallidus [54], the rostral medullary brainstem [55], and thalamus [56], here we focus on the important role of the VLPO [57] and note that this component of the model could in principle represent one or more sleep-promoting centers that act in concert.

The dynamics of sleep and wake are thought to be controlled primarily by the circadian, 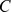, and homeostatic, 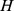, drives [23], [25]. The 24 h periodic circadian signal, which originates in the suprachiasmatic nucleus of the hypothalamus (SCN), is entrained by the light/dark cycle [4]. The VLPO receives an inhibitory circadian projection, while Orx receives an excitatory circadian projection, primarily via the dorsomedial nucleus of the hypothalamus (DMH) [4], [58], [59]. The homeostatic sleep drive, 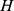, increases during wake and decreases during sleep, and may correspond to some sleep-regulatory substance [60], [61], such as adenosine [62], [63] or cytokines [36], [64]. The homeostatic sleep drive disinhibits the VLPO [4], [52].

**Figure 1:**
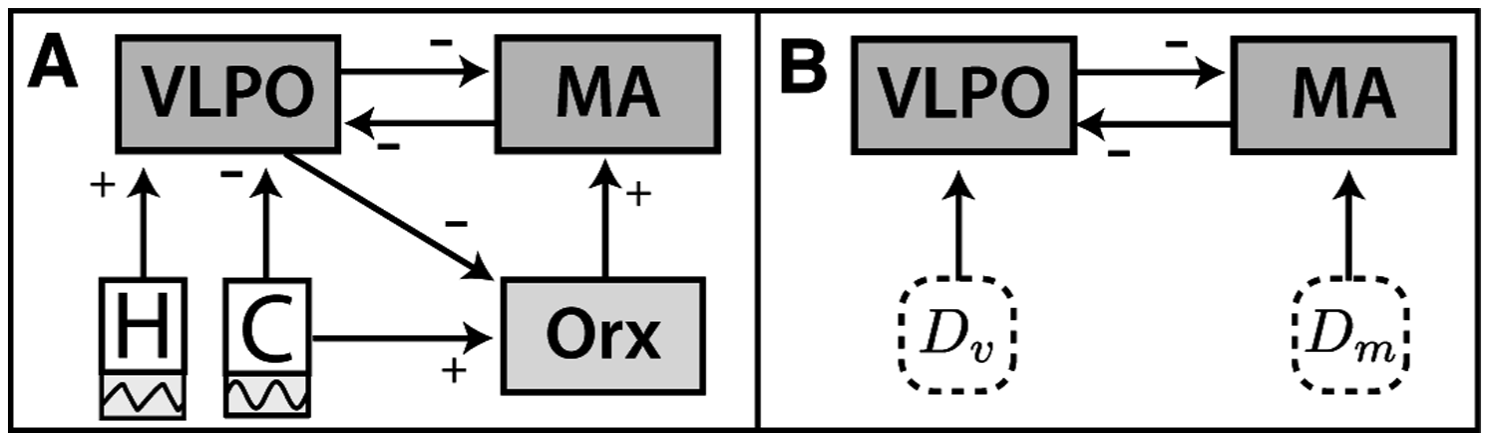
Schematic of the model. The model includes interactions between the sleep-active ventrolateral preoptic area of the hypothalamus (VLPO), the wake-active monoaminergic brainstem nuclei (MA), and the orexinergic neurons of the lateral hypothalamic area (Orx), as well as the circadian (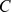) and homeostatic (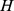) drives. Arrows indicate interactions between the populations, as well as the pathways of the circadian and homeostatic drives, and represent either excitatory (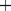) or inhibitory (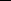) interactions. **A** All modeled interactions are shown, including the mutual inhibition between VLPO and MA [27], inhibition of Orx by VLPO [8], and excitatory input from Orx to MA [72]. The circadian drive, 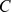, which originates in the suprachiasmatic nucleus (SCN), is afferent to both VLPO (inhibition) and Orx (excitation) [4], while the homeostatic sleep drive, 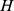, which increases during wake and decreases during sleep, disinhibits VLPO [63]. Example two day time traces for normal sleep-wake behavior are annotated below the 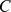 and 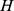 drives. **B** The model can be mathematically reduced to the core dynamics of mutual inhibition between the sleep-active VLPO and wake-active MA groups. In this representation, net drives, 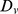 and 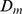, to VLPO and MA, respectively, control the arousal state dynamics. This reduced representation is used throughout this work to visualize and understand the model dynamics.

A schematic depiction of the model, which includes the neuronal interactions and drives described above, is shown in Fig. 1A. For analytical purposes, the model can be analyzed in a reduced representation that focuses on the MA–VLPO sleep-wake switch, as shown in Fig. 1B. In this picture, net external drives to the VLPO and MA are grouped as 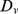 and 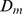, respectively, and control the evolution of arousal state over time: 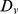 includes inhibition from 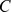 and disinhibition from 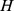, while 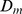 includes an excitatory input from Orx, which itself receives an excitatory input from 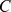. This reduced representation is used in this work help explain how Orx acts to stabilize sleep and wake by modulating 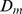. The remainder of this section contains details of how the neuronal populations and drives described above are modeled mathematically.

### Neuronal Interactions

Our model captures the average properties of populations of neurons and their interactions [65], and is based on previously successful approaches to modeling the corticothalamic system [65]–[67]. Each population, 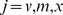, where 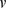 stands for VLPO, 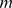 for MA, and 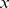 for Orx, is represented by its mean cell-body potential relative to resting, 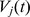. The mean firing rate of each population, 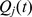, is approximated by a sigmoidal function of 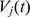 [68]:
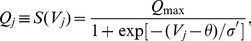
(1)where 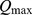 is the maximum possible firing rate, 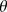 is the mean firing threshold relative to resting, and 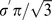 is its standard deviation [66]. Due to the small volume of the relevant nuclei, we assume spatial homogeneity of each population and neglect propagation delays between neurons. This assumption is reasonable because interactions within the relevant neuronal populations occur on timescales of milliseconds, whereas we are interested in capturing arousal-state dynamics that occur on timescales of seconds or longer. We assume that changes in postsynaptic potentials are proportional to the firing rates, 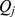, of the presynaptic populations, and use the constants, 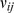, to represent the strength of the synaptic connection from population 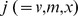 to population 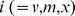. Time constants, 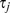, control the rate at which the dynamics of 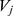 evolve via the decay rate of neuromodulator effects.

The equations governing the VLPO (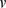) and MA (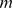) populations are as follows:
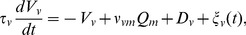
(2)




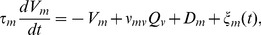
(3)where the negative coefficients, 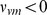 and 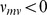, capture the mutual inhibition of the two populations [27], [46]. Net drives are grouped as 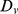 and 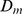, while 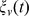 and 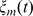 are independent, Gaussian-distributed, zero-mean white noise processes with standard deviations 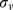 and 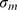, respectively. These noise variables represent the inherent noise in biological processes and fluctuating external inputs to these populations. In the absence of physiological data to estimate the relative noisiness of drives to the VLPO and MA, we set them equal here for simplicity (i.e., 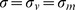) so that 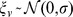 and 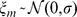. Equations (2) and (3) thus capture the flip-flop dynamics between the VLPO and MA [27].

Orx is modeled as a neuronal population in the same way as for the MA and VLPO, with dynamics governed by.
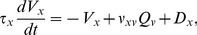
(4)where 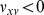 captures the inhibition of Orx by the VLPO [8], [69], and drives to Orx are grouped as 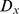. Due to inhibition from the VLPO, Orx is suppressed during sleep, but is active during wake when the VLPO is inactive. Orx may receive inhibitory inputs from serotonin and norepinephrine [50], while noradrenergic input has been shown to be excitatory, but inhibitory following sleep deprivation [70]; other studies have reported no reciprocal connections from monoamine-containing groups that are innervated by Orx [71]. Given this uncertainty in the net connection between MA and Orx, we assume it to be small relative to the other terms modeled in Eq. (4) and neglect it by setting 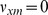 here. We note that a large positive 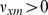 could produce an instability in the model with mutually-excitatory Orx and MA reinforcing the behavior of each other during wake (that would require the modeling of additional systems to stabilize), while small negative connections, 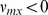, could be accommodated with relatively minimal affect on the qualitative dynamics reported here [e.g., compensating by increasing both 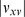 and 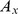, cf. Eq. (9)]. A noisy input to Orx is not included for simplicity, because Orx excites the MA in the model, which itself receives a direct noisy input, 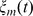. If modeled, input noise to Orx, 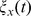, added to Eq. (4), would be relayed to the MA during wake when Orx is active, but suppressed during sleep when Orx is inactive, producing noisier waking periods, but otherwise having a minimal effect on the model dynamics.

### Drives

The circadian drive for wake is taken to be entrained to the daily light/dark cycle and is approximated by a sinusoidal function of time,
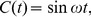
(5)where 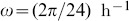. An oscillation amplitude of unity is used without loss of generality because the actual amplitude is absorbed into the weights, 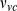 and 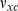, that control the circadian inputs to VLPO and Orx, respectively, while any constant offsets in 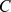 are incorporated in the constants 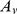 and 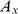 in Eqs (7) and (9), respectively. This sinusoidal form of 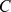 is used here for simplicity, but we note that dynamic circadian oscillator models can also be implemented straightforwardly [42], [43].

The dynamics of the homeostatic sleep drive, 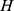, depend on the arousal state, as described above. Our model of 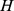 is based on a sleep-promoting factor that increases during wake when the MA is active (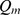 is high) and decreases during sleep when the MA is suppressed (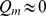). These dynamics are described by
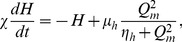
(6)where 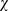 sets the timescale on which 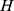 changes, and the constants 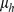 and 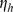 control the dependence of 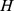 production on 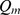. In some previous work, a linear 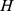 production term was used [35], which is appropriate for modeling basic sleep-wake dynamics when 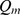 does not vary significantly during wake. In this work, however, waking arousal states with very different 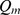 are simulated, and thus the saturating form of the final term in Eq. (6) (introduced previously [38]) is required to avoid unreasonably large disparities in 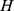 production between waking states with different 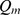.

Net drives to each neuronal population are defined as follows:
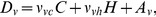
(7)

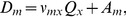
(8)

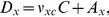
(9)where 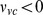 because the VLPO receives an inhibitory projection from the DMH, which itself receives an excitatory circadian projection [4], [59]; 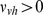 because the homeostatic process 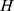 disinhibits the VLPO [4], [52], [63]; 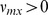 because Orx excites the MA [72] (including the DR [73], LC [74], and TMN [75]); and 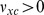 because Orx receives a strong excitatory circadian projection from the DMH [4], [58]. Inhibition of Orx by homeostatic sleep-regulatory substances [76] is assumed to be small and is neglected here by setting 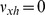. Constants, 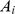, represent time-averaged inputs to each population from external sources not explicitly modeled here, and could include any constant offsets of the circadian drive for 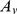 and 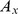, or any time-averaged cholinergic inputs to the MA for 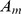, for example. Altogether, 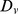 includes inhibition from 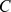 and disinhibition from 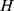, 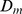 varies with Orx activity, and 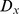 is circadian. The excitation of MA by Orx is included as the 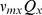 term in 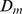 [Eq. (8)] rather than appearing directly in Eq. (3), which is mathematically equivalent but allows us to focus on the sleep-wake switch by interpreting Orx as a component of 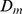. This excitatory input to the MA is the only effect of Orx on the rest of the model, and hence Orx loss can be simulated by reducing the single parameter 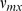. Finally note that because the drive to Orx, 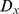, affects the sleep-wake switch only through 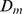, we can focus on a reduced form of the model in which the dynamics are summarized by the values of 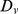 and 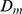, as in Fig. 1B. This approximation is valid if 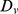 and 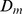 are slowly-varying compared to the dynamics of 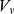 and 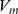, which holds here because 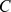 and 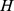 vary on the timescale of a day, and 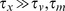.

### Parameter Constraints and Computation

Compared to the original Phillips-Robinson model, the current model includes new parameters, as well as adjustments to some existing parameters, as listed in Table 1. Most existing parameters are unchanged, with new and altered parameters shown in boldface in Table 1. We maintain as much compatibility with previous work as possible to ensure that previous model predictions are retained and that changes to the model’s structure represent improvements rather than simply providing more flexibility to fit the phenomena presented here. For example, the new model reproduces both the normal flip-flop sleep-wake dynamics of the Phillips-Robinson model, and the same behavior during total sleep deprivation as reported previously [38] (see File S1).

**Table 1:** Nominal model parameter values.

	Param.	Value	Param.	Value	Param.	Value
Connection strengths	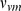	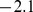 mV s	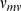	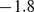 mV s	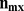	**0.3 mV s**
	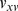	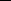 **1.0 mV s**				
Time constants	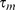	10 s	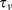	10 s	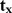	**120 s**
Sigmoid parameters	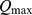	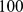 s 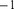	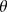	10 mV	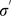	3 mV
Drive parameters	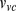	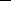 **0.30 mV s**	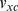	**1.0 mV s**	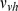	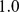 mV
	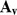	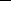 **8.5 mV**	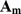	**0.52 mV**	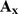	**1.0 mV**
Homeostatic dynamics	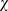	45 h	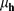	**17 s**	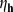	**2.3**
Noise standard deviation	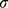	**1 mV**				

Parameters either introduced or modified in this work are shown in boldface. All other parameter values correspond to those of the original Phillips-Robinson model, which were constrained and subsequently verified on a broad range of experimental protocols including sleep deprivation [37], [38], sleep fragmentation [39], caffeine intake [40], mammalian sleep [41], shift work [42], and internal desynchrony [43] in previous work. New parameters introduced here are 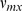, 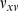, 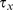, 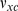, 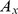, and 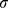.

Parameters are constrained separately in different dynamical regimes of the model. With Orx absent from the model, the qualitative dynamics should reflect a severe narcoleptic or Orx-knockout phenotype, which we use to constrain the constant inputs, 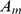 and 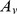, the circadian parameters, 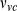 and 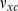, and the noise variance, 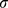. The homeostatic production parameters, 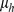 and 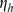, are set to maintain approximately eight hour daily sleep durations across a range of 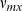; 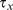 is set to match the empirical timescale of sleep inertia (explained in detail later); and the Orx parameters, 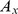, 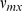, and 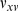, are set to maintain normal sleep-wake behavior. Further details of how the parameters are constrained, including justifications for all parameter values, are in File S1. Note that the aim of this study is not to perform rigorous parameter constraints by fitting to clinical datasets (which could be performed in future), but rather to show that physiologically reasonable values of parameters exist that can plausibly account for clinical observations of narcolepsy.

The current model differs from the original Phillips-Robinson model [35] in two key ways. Most obviously, the new model includes Orx [Eq. (4)], which contributes a time-varying drive to the MA that was previously constant (the parameter 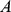 in the original model). The other major change is the reduced magnitude of circadian input to the VLPO, 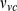. In the Phillips-Robinson model, the circadian drive affected the sleep-wake switch only as an input to VLPO, with 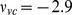 mV [35]. However, following VLPO lesions, strong circadian rhythmicity in sleep-wake behavior persists [57], and Orx has been shown to play an important role in the circadian control of sleep [77]. These experimental results suggest that the dominant circadian input to the sleep-wake switch may be via Orx to MA. The parameters used in this model reflect this, with 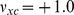 mV s and 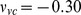 mV s (cf. File S1). In future work, physiological and behavioral data could be used to further constrain the relative contributions of these two circadian pathways (changes in which have been shown to generate sleep phenotypes of other mammalian species [78]).

Combining the definitions of the neuronal interactions and drives above, the output of the full model is the solution of the following four coupled differential equations:
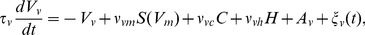
(10)

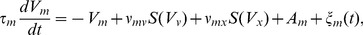
(11)

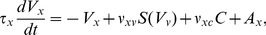
(12)

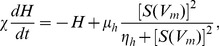
(13)where 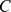 is defined in Eq. (5). When noise is included in the model, these stochastic differential equations are solved numerically using the Euler-Marayama method [79] with a time step 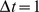 s (time steps 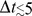 s produced sufficiently converged dynamics). The model can be simulated without noise by removing the terms 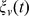 and 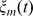 from Eqs (10) and (11), whence the model equations reduce to four coupled ordinary differential equations that are solved numerically using the variable-order solver for stiff problems, **ode23s**, in Matlab 2011b (Matlab is a product of The MathWorks, Natick, MA). Throughout this work, periods in which 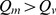 are labeled ‘wake’ and periods in which 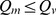 are labeled ‘sleep’; transient noisy fluctuations in state lasting less than 60 s are ignored (the main results are not sensitive to this state-labeling heuristic, see File S1).

## Results and Discussion

In this section a detailed analysis of the model is used to characterize Orx’s role in sleep-wake dynamics. First we investigate the model’s dynamical properties in terms of the net drives to the sleep-active VLPO and wake-active MA: 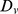 and 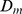, respectively. The results are used to explain how the loss of Orx in the model reduces waking arousal and lowers thresholds for transitions between wake and sleep, as occurs in narcolepsy. Simulations indeed reveal an increase in sleep-wake fragmentation as orexin levels are reduced, as well as changes to a range of other key sleep-wake statistics. Finally, we explain how dynamics resembling sleep inertia are predicted by the model due to an asymmetry between sleep-to-wake and wake-to-sleep transitions.

### Dependence of Sleep-wake Dynamics on Net Drives to the VLPO and MA

In this section, we explain how the model’s dynamics depend on the net drives to the sleep-wake switch: 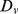 and 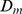. In particular, we identify combinations of 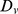 and 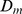 that produce: (i) a stable wake state, (ii) a stable sleep state, and (iii) where wake and sleep are simultaneously stable and noise-induced transitions between the two states are possible. The analysis will facilitate an understanding of the full model dynamics, which will be investigated in later sections. Note that the results of this section hold equally for the current model and the original Phillips-Robinson model [35], which was also centered around the VLPO–MA sleep-wake switch, because the parameters that determine the dynamical properties of this space: 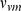 and 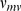, and the sigmoidal function [Eq. (1)], are not altered in this work.

**Figure 2:**
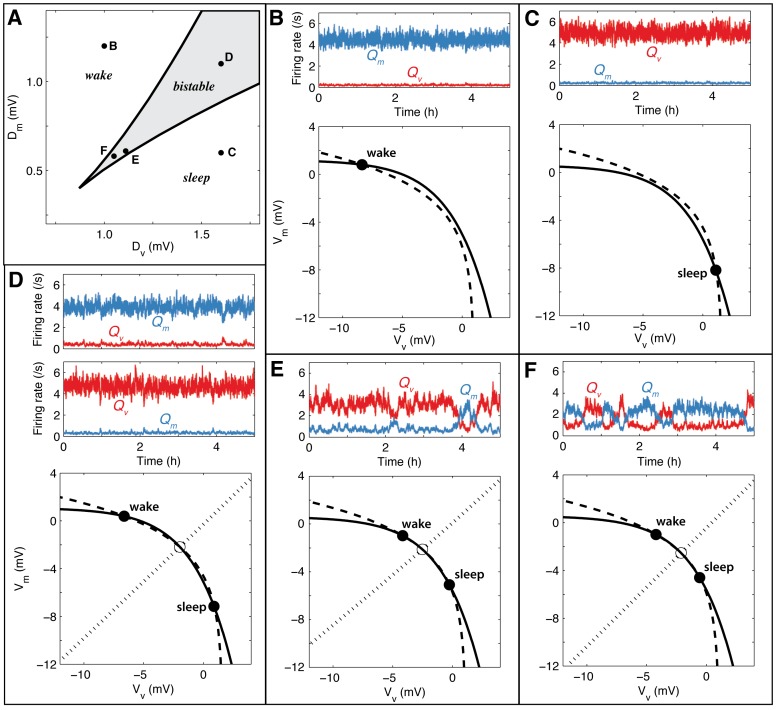
Model dynamics represented in terms of the net drives to the sleep-active VLPO, 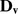, and the wake-active MA, 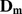. **A** Three distinct regions of 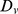
–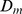 space are: (i) *wake*: at low 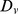 and high 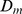 a stable wake state exists, (ii) *sleep*: at high 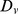 and low 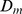 a stable sleep state exists, and (iii) *bistable* (shaded): at intermediate 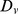 and 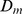 wake and sleep states are simultaneously stable and transient noise can produce lasting changes of state. Simulated 5-h time series and 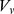
–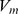 plots for fixed points in this space are shown in the remaining figures. Time series are plotted for average firing rates of the VLPO, 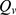 (red), and the MA, 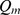 (blue). In the 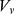
–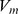 plots, we include 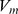 nullclines (solid lines), 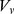 nullclines (dashed lines), stable equilibriums (solid circles), saddle points (open circles), and the separatrix (dotted black line); see File S1 for definitions and numerical details. **B** 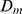 is high and 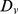 is low; a single stable wake state exists. **C** 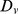 is high and 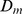 is low; a single stable sleep state exists. **D** In the bistable region at high 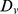 and 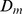, thresholds for transitions between wake and sleep are high and hence state transitions are extremely improbable: the system remains either awake or asleep depending on its initial state (on timescales relevant to the current dynamics). **E** In the bistable region nearer the sleep bifurcation boundary, transitions from wake to sleep are more probable than transitions from sleep to wake. **F** In the bistable region at low 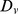 and 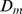, thresholds for transitions between sleep and wake are low and simulated time series are highly fragmented.

A reduced representation of the model, in terms of the net drives 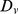 and 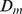, is shown schematically in Fig. 1B. In Fig. 2A, the model’s equilibrium states are labeled in this space and, as might be expected intuitively, increasing 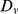 promotes sleep and increasing 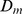 promotes wake. Importantly, we find an intermediate set of drives, 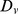 and 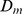, for which sleep and wake states are simultaneously stable (the bistable region shaded in Fig. 2A). Model dynamics at fixed values of 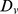 and 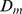 are represented in the space of the average cell-body potentials of the VLPO, 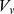, and the MA, 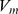, as shown in the remaining 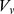
–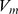 plots in Figs. 2B–F, for selected values of 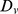 and 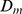. The 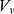
–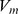 plot was introduced in previous work to analyze the model on timescales shorter than that of changes in 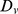 and 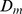, which can be treated as control parameters of the fast dynamics [39].

The system is awake at high 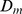 and low 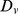: the region labeled ‘wake’ in Fig. 2A. In this region, the system attracts onto a single stable equilibrium that corresponds to a waking state with high 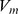 and low 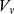 (i.e., active MA and suppressed VLPO). The drives, 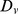 and 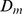, control the level of waking arousal in this region: 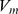 (and hence 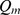) increases with 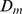 (higher drive to wake) and decreases with 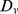 (higher drive to sleep), and vice-versa for 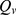. An example 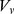
–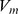 representation of the model in this waking region, at 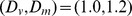 mV, is shown in Fig. 2B. Example time series for 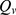 and 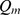 at these net drives with noise, plotted in the upper panel of Fig. 2B, are the result of noisy deviations from the stable waking state, combined with the attraction of the system back toward equilibrium.

At high 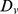 and low 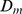, the region labeled ‘sleep’ in Fig. 2A, the system attracts onto a single stable equilibrium, as above, but now the equilibrium is a sleep state with active VLPO and suppressed MA. As before, the steady-state firing rate, 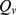, of the sleep equilibrium increases with 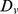 and decreases with 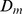, and vice-versa for 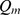. An example is given for 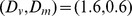 mV in Fig. 2C. Dynamics consist of noisy perturbations about the stable sleep state.

More complex dynamics occur at intermediate 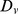 and 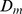: the shaded bistable region in Fig. 2A. The boundaries of this bistable region correspond to saddle-node bifurcations of the model [39] (see File S1 for mathematical details). We refer to the leftmost boundary in Fig. 2A as the ‘wake bifurcation boundary’ (beyond which only wake is stable), and the rightmost boundary as the ‘sleep bifurcation boundary’ (beyond which only sleep is stable). In the bistable region, the stable wake and sleep equilibriums coexist, and are separated by a separatrix in 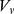
–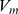 space, which is plotted as a dotted line in Figs. 2D–F. The two regions on either side of this separatrix correspond to wake and sleep basins: when the system is in the wake basin it will attract (deterministically) onto the stable wake equilibrium and when it is in the sleep basin it will attract (deterministically) onto the stable sleep equilibrium. Transient external drives can cause the system to cross this separatrix and thereby change state. We consider only the noise processes 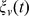 and 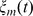 in this work, but note that other types of impulsive drives could also cause a lasting change in the state of the system, e.g., the short acoustic stimuli during sleep modeled in previous work [39]. Three points in the bistable region, labeled D, E, and F in Fig. 2A are shown in Figs. 2D–F and will be studied in turn.

In the bistable region with 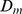 and 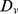 both high, e.g., for 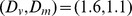 mV, shown in Fig. 2D, the sleep and wake equilibriums are well-separated and the thresholds for transitioning between sleep and wake are high. Consequently, state transitions are highly improbable, and the system mostly acts as if only a single stable equilibrium exists: remaining either awake or asleep depending on its initial condition. Time series are shown in Fig. 2D for when the system is initially in a wake state, and when the system is initially in a sleep state. In both cases, noise with 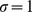 mV is insufficient to change the state of the system (on timescales meaningful to the current dynamics).

In the bistable region at lower net drives, 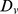 and 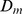, the thresholds for state transitions decrease so that noise can change the state of the system. The probabilities of wake-to-sleep and sleep-to-wake transitions depend on the position in the bistable region, and are in general unequal. As the system approaches the sleep bifurcation boundary, the wake equilibrium moves closer to the saddle point and the sleep equilibrium moves further from the saddle point, thereby biasing the transition probabilities further toward sleep. The reverse occurs near the wake bifurcation boundary, where sleep-to-wake transitions become increasingly more probable than wake-to-sleep transitions. For example, consider the point 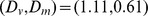 mV, labeled ‘E’ in Fig. 2A, which is nearer the sleep bifurcation boundary than the wake bifurcation boundary. Here, thresholds for state transitions are relatively low and the position of the wake equilibrium is closer to the saddle point than the position of the sleep equilibrium, as shown in Fig. 2E. Wake-to-sleep transitions are more probable than sleep-to-wake transitions, and simulated time series, such as that plotted in Fig. 2E, show the system mostly in sustained sleep periods, while wake bouts are relatively short-lived.

Finally, we study the model dynamics at very low drives, 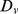 and 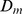, in the bistable region, using the point 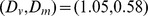 mV as an example, shown in Fig. 2F. In this region, the stable sleep and wake equilibriums are both close to the saddle point so that thresholds for state transitions are very low and hence state transitions are highly probable with noise in the model. An example time series generated at these net drives, shown in the upper panel of Fig. 2F, is highly fragmented, with frequent transitions between sleep and wake. Equilibrium mean firing rates are relatively low: both 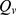 during sleep, 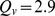 s^−1^, and 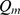 during wake, 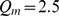 s^−1^, indicative of a weakening of the normally pronounced sleep-wake distinction.

In summary, we have shown that lasting transitions between sleep and wake can only occur for a subset of drives, 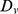 and 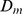, in the bistable region, with state transition thresholds that decrease as 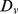 and 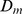 decrease. Note that at low 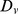 and 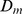 beyond the bistable region (i.e., the lower lefthand corner of Fig. 2A), mean firing rates of both populations are low; this pathological regime is not accessible for the parameters used in the current model formulation (without adding persistent external drives).

### The Effect of Orx on Thresholds for State Transitions

In this section, the above characterization of the model’s dynamics as a function of 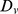 and 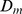 is used to understand how time-varying inputs to both populations control the evolution of arousal-state dynamics. As explained in *Models* above, the net drive to VLPO, 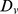 [Eq. (7)], includes an oscillatory circadian input, 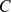, a homeostatic sleep drive, 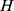, that increases during wake and decreases during sleep, and other constant drives, 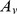. The net drive to MA, 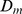 [Eq. (8)], includes an excitatory input from Orx, 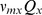, and time-averaged drives from processes not modeled here, 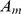. Each of these physiological mechanisms contributes to moving the system through the 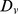
–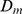 plane and their combination determines the arousal-state dynamics of the model. The model is examined *without* added noise in this section to provide a preliminary understanding of the regions of the 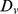
–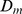 plane that the system moves through; the role of noise in producing state transitions is investigated later.

**Figure 3:**
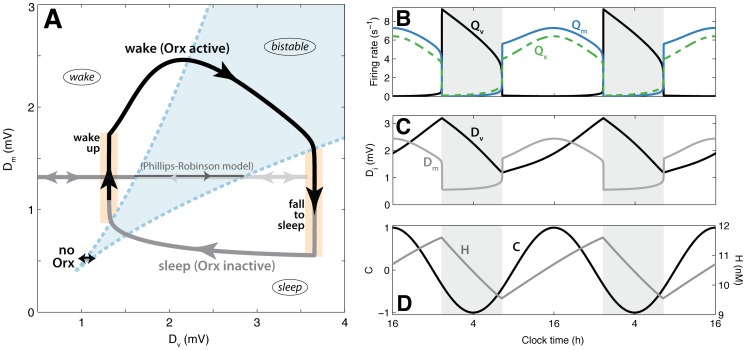
Noise-free model simulations represented as trajectories in terms of net drives to the VLPO, 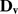, and MA, 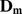, and as time series. **A** The bistable region is shaded blue, and the wake and sleep regions are labeled. The trajectory for normal dynamics (i.e., including Orx) forms a loop and is plotted using black (wake) and gray (sleep). The trajectory for the model without Orx is a small oscillation at low 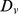 and 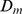, and is labeled ‘no Orx’. The trajectory for the original Phillips-Robinson model occurs at fixed 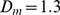 mV and is shown semi-transparent for comparison (note that the wake trajectory of the Phillips-Robinson model extends beyond the limits of the figure to 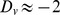 mV). When Orx is removed from the model, the system oscillates at low 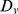 and 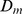, where thresholds for transitions between wake and sleep are low. However, with Orx in the model, the wake and sleep states are stabilized: Orx is active during wake, increasing 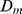, and Orx is suppressed during sleep, decreasing 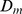, thereby moving the system away from the bistable region where state transitions can occur and promoting consolidated wake and sleep episodes. Circadian input to Orx modulates waking arousal levels: 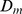 is lower in the early morning and increases to a maximum at the circadian maximum, then decreases through the afternoon and evening. Two-day time series for noise-free model dynamics (including Orx) are also plotted as: **B** Firing rates 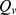 (black), 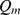 (blue), and 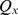 (green, dashed), **C** Net drives to the VLPO, 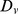 [black, Eq. (7)], and the MA, 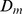 [gray, Eq. (8)], and **D** Drives 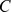 [black, Eq. (5)] and 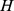 [gray, Eq. (6)]. Approximate clock times for a typical sleep-wake schedule are given as a guide, and sleep periods are shaded.

For reference, we first describe the original Phillips-Robinson model [35], in which 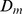 is constant and trajectories in the 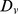
–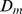 plane are horizontal lines. The combination of 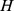 and 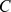 provides a net oscillatory drive, 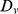, producing normal, flip-flop sleep-wake dynamics, as shown in Fig. 3A. Due to the 24 h oscillation in 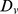, the system is driven back and forth between sleep and wake, falling asleep at the sleep bifurcation boundary at high 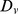 and waking up at the wake bifurcation boundary at a lower 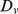. Because 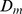 is constant, the Phillips-Robinson model can be represented as a function of the single control parameter 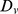 (on timescales shorter than that of changes in 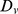 [39]), yielding a hysteresis loop [35], [38].

With Orx included, the new model produces a loop-like trajectory through the 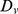
–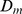 plane, shown in Fig. 3A. When the system is asleep (plotted gray in Fig. 3A), Orx is inactive (i.e., 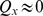), due to inhibition from the VLPO, and 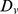 decreases, mostly due to a decreasing homeostatic sleep drive, 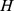. When the system wakes up, Orx activates, exciting the MA and causing an increase in 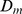 that moves the system out of the bistable region where transitions between wake and sleep can occur. During the waking period (plotted black in Fig. 3A), 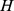 builds, increasing 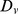, and the excitatory circadian input to Orx modulates waking arousal levels, increasing 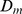 (and hence 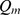) to a maximum at the circadian peak. The system then moves rapidly through the bistable region during the evening, with increasing 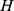 and decreasing 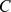 moving the trajectory downwards and to the right, eventually to a sufficiently low 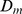 that a transition back to sleep occurs. After the system has fallen asleep, VLPO activates and suppresses Orx, reducing 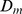 and preventing transitions back to wake, thus facilitating another consolidated sleep bout. That Orx activates during wake and is suppressed during sleep therefore moves the system away from the bistable region where transitions can occur and promotes consolidated bouts of both sleep and wake.

Note that external influences, such as intense physical activity or caffeine at the end of the day would contribute an arousing drive and increase 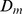 (perhaps directly [80], and/or via Orx [81]), and thereby prolong wake. Conversely, lying in bed in a dark room would reduce the net input to the MA from arousing sensory stimuli and decrease 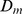, hastening the transition to sleep. These examples help to demonstrate how the model could be applied to intuitive real-world scenarios with more complicated environmental stimuli, but we do not pursue them further here.

Because Orx enters our model as an excitatory input to MA, we can investigate model dynamics with Orx completely removed from the model by setting 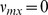. This yields the small trajectory labeled ‘no Orx’ in Fig. 3A. The trajectory occurs at a constant 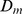 (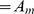), and oscillates horizontally according to the homeostatic and circadian components of 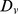 [Eq. (7)]. As explained above, thresholds for state transitions in the bistable region at low 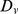 and 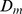 are very low and waking arousal, 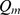, is reduced, as is 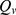 during sleep (cf. Fig. 2F). This region of the drive space, which results from eliminating Orx from our model, thus characterizes many of the known properties of the narcoleptic phenotype: low thresholds for transitions between states and low waking arousal. In simulations below, we will show that when noise is added to the model, the dynamics of sleep and wake are correspondingly fragmented.

Thus, the model predicts three key mechanisms through which Orx acts to stabilize prolonged sleep and wake episodes: (i) Orx excites the MA during wake, increasing 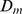, enhancing waking arousal levels, 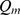, and raising the threshold for transitions to sleep during a wake episode, (ii) Orx is suppressed by the VLPO during sleep, decreasing 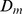 and preventing transitions back to wake, and (iii) The excitatory circadian drive to Orx is relayed to the MA during wake, further stabilizing wake during waking circadian phases. Orx therefore stabilizes *both* wake (by increasing 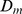 during wake) *and* sleep (by decreasing 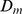 during sleep).

Two-day time series for 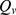, 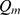, and 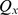, generated by the noise-free model (including Orx) are plotted in Fig. 3B. During sleep, 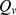 is high and decreases across the night until the transition to wake, during which Orx relays a circadian variation in waking arousal levels, which peaks with the circadian drive near the middle of the wake episode (cf. Fig. 3D). As shown in Fig. 3C, 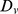 is dominated by 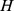, which decreases during sleep and increases during wake. The net drive to MA, 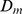, is low during sleep and high during wake, reflecting Orx activity. Notice that the circadian input to Orx has a negligible effect on the system during sleep when Orx is suppressed, but plays an important role in modulating Orx (and hence MA) activity during wake. Time series for 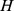 and 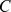 are plotted in Fig. 3D for comparison.

### Simulating Narcolepsy

**Figure 4:**
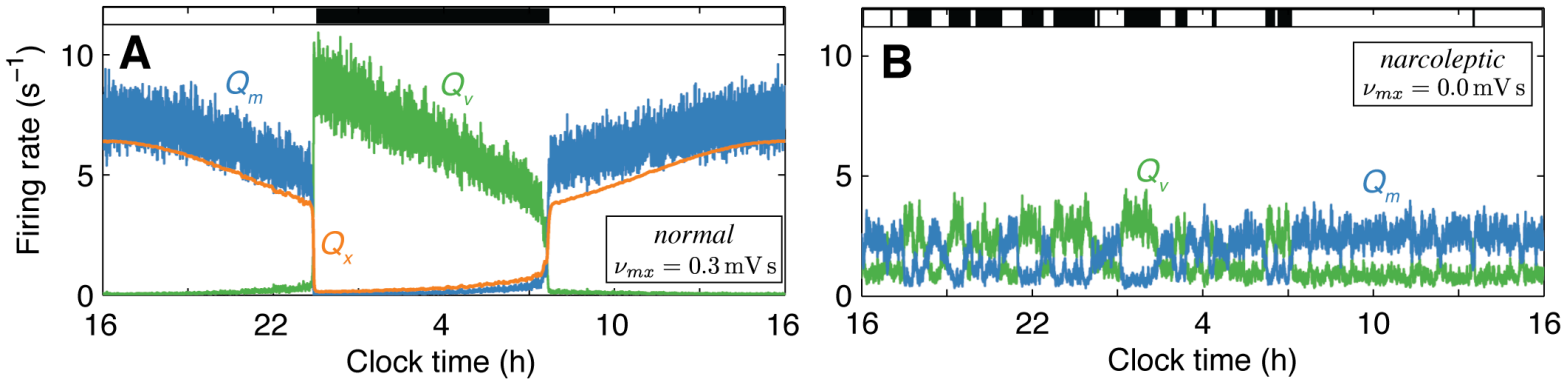
Removing Orx from the model produces fragmented sleep-wake time series characteristic of the narcoleptic phenotype. Simulated 24-h time series are plotted for **A** Normal dynamics including Orx (i.e., 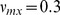 mV s) for 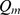 (blue), 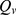 (green), and 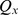 (orange), and **B** Fragmented dynamics with Orx removed from the model (i.e., 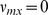). Periods of sleep, with 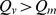 (black), and wake, with 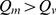 (white), are shown in the strip above the main plot. When 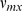 is reduced, the system moves from a regime in which Orx stabilizes extended wake and sleep bouts, to a regime characterized by low waking arousal levels and increased fragmentation due to a lowering of the threshold for state transitions.

In this section, we include noise in the model and use simulations to explain how the loss of Orx leads to the behavioral state instability that characterizes narcolepsy. As described above, because Orx enters our model as an excitatory input to MA, Orx loss can be simulated by reducing 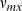. Note that here we simulate a total loss of Orx by setting 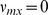, as an Orx knockout or severe narcoleptic, rather than the approximately 90% reduction that occurs in narcolepsy (i.e., to 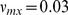 mV s) to simplify the analysis; the difference is small and a detailed investigation into the dependence of the dynamics on 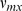 is provided below. Simulated 24 h firing rate time series are plotted for normal sleep-wake behavior, with 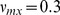 mV s, in Fig. 4A, and with 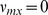 in Fig. 4B. No other parameters were altered between these simulations.

The model’s outputs for normal sleep-wake behavior (in Fig. 4) resemble those shown above for the noise-free case, with Orx stabilizing extended daily wake and sleep episodes. With noise in the model, sleep becomes viable toward the end of the evening (cf. Fig. 3A), where the threshold for a transition to sleep decreases, allowing external influences (noise in this simulation), to determine the precise timing of the wake-to-sleep transition. The model produces realistic firing rates, both in terms of their magnitudes and temporal organization, for all populations. For example, physiological data suggest that 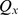 is approximately 4–8 s^−1^ during wake and 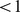 s^−1^ during sleep [81], [82]; the model has 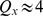
–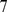 s^−1^ during wake and 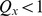 s^−1^ during sleep, while firing rates for MA and VLPO are similar to those produced by the original Phillips-Robinson model.

Simulated 24 h firing rate time series with Orx absent from the model (i.e., 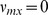) are plotted in Fig. 4B. As explained above, thresholds for state transitions at low 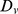 and 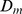 in the bistable region are very low, with noise causing frequent state transitions. Without Orx to increase 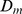 and stabilize wake, or decrease 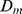 to stabilize sleep, the system no longer has a mechanism for producing extended episodes of either wake or sleep. When the system is asleep, 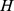 decreases, pushing the system to lower 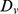 where transitions to wake are more probable. Conversely, when the system is awake, 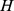 increases and pushes the system to higher 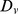, where transitions to sleep are more probable. The system is unable to escape this cycle of severe sleep-wake fragmentation. Circadian input to the VLPO adds a circadian phase dependence to the probability of the system being awake (higher at higher 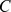) or asleep (higher at lower 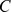). Waking arousal levels (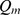) are reduced compared to normal individuals because Orx increases 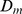 during wake, a mechanism that is absent without Orx. Two main consequences of decreasing 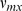 in our model are therefore lower waking 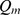, and lower thresholds for state transitions, corresponding to two key features of the narcoleptic phenotype.

### Dependence of Sleep-wake Dynamics on Orexin Levels

**Figure 5:**
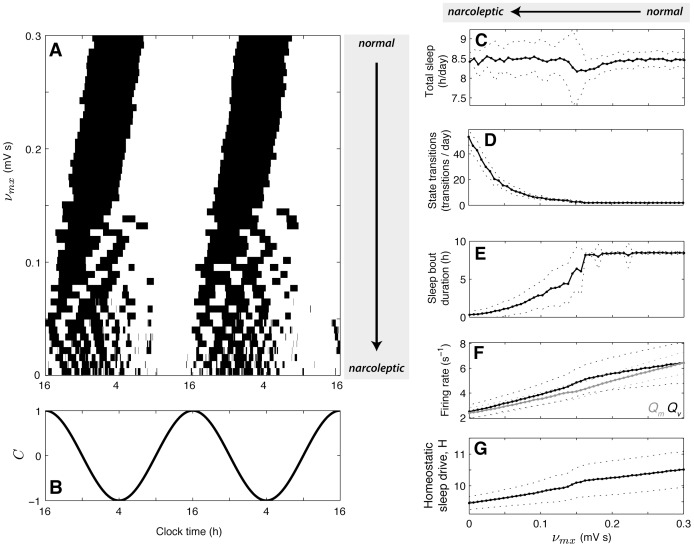
Model dynamics as a function of orexin levels, corresponding to the model parameter 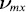. **A** Periods of sleep (black) and wake (white) are plotted as a function of 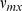 across two-day model simulations. **B** The circadian drive, 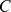, versus time. Various statistics taken from the model output are plotted as a function of 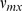 as the mean (solid) 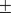 standard deviation (dotted) measured across a 25 day model simulation (following a 3 day equilibration period), for **C** Total sleep duration per day, **D** Number of state transitions per day, **E** Duration of sleep bouts, **F** 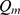 during wake (blue) and 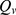 during sleep (black), and **G** Homeostatic sleep drive, 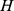.

In this section, we explore how simulated sleep-wake dynamics depend on 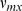 across the full range 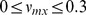 mV s. Results are shown in Fig. 5. In Fig. 5A, the gradual increase in sleep-wake fragmentation that results from decreasing 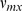 in our model is shown for two-day simulations by plotting sleep (black) and wake (white) periods. Prior to each simulation, the model was equilibrated by simulating it for three days at each given 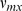. As 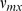 decreases down to a reduction of approximately 50% (i.e., 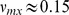 mV s), consolidated bouts of sleep and wake are still possible, but with sleep periods commencing at an earlier circadian phase (i.e., morningness). The model thus predicts that consolidated sleep is robust to modest differences in orexin levels, but also that these differences may contribute to differences in chronotype. This represents an important potential addition to the list of factors that are already known to influence chronotype: inter-individual differences in patterns of self-selected light exposure, and differences in circadian and homeostatic processes [83]. This observation may also offer a potential explanation for the tendency to morningness and more fragmented sleep with aging [84], as orexin levels gradually decline [85]. The predicted phase advance of sleep with reduction in orexin levels should be investigated further–if borne out in clinical experiments, it may also have therapeutic value for early disease detection, for example.

At more severe levels of Orx loss (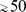
% reduction), the system can no longer sustain extended bouts of sleep and wake. Through the mechanisms explained in the preceding sections, sleep-wake fragmentation increases as 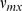 decreases: both naps during normal wake periods, and awakenings during normal sleep periods. Figure 5A reveals sharp wake-to-sleep transitions, but more volatile sleep-to-wake transitions (often exhibiting ‘snoozing’ back to sleep, for example); this asymmetry is related to the role of Orx in sleep inertia, and is characterized in detail below. The two day time series for the circadian drive, 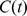, in Fig. 5B, reveals a strong circadian phase dependence of sleep and wake across a wide range of 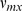.

The model predicts that decreasing orexin levels affects a range of relevant sleep-wake statistics. A selection of summary statistics of the model’s output across 25 day model simulations are shown in Figs. 5C–G at each of 51 equally-spaced points for 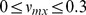 mV s. Figure 5C shows that the total sleep time remains approximately constant with changes in 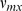, a feature that is observed clinically [86] and was used to fit the model (see File S1). As shown in Fig. 5D, the number of state transitions per day remains at two (one sleep and one wake transition) when 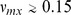 mV s, then increases smoothly as 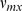 decreases, to approximately 53 per day when 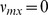. The mean duration of sleep bouts correspondingly decreases for 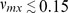 mV s, as plotted in Fig. 5E. The firing rates, 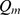 during wake, and 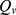 during sleep, shown in Fig. 5F, decrease as 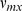 decreases, due to reduced promotion of wake. As shown in Fig. 5G, the mean value of 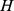 decreases slightly as 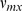 is decreased, from 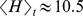 for 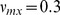 mV s (normals) to 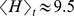 for 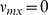.

Note that only the single model parameter, 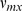, was altered in these simulations; the circadian and homeostatic drives were not changed, consistent with available experimental evidence suggesting that the circadian and homeostatic processes themselves appear to be normal in narcoleptics [13], [19]. We also emphasize that our aim is to show the qualitative behavior of our model as orexin levels decrease, while the quantitative values predicted could be fitted to specific clinical datasets in the future.

### Sleep Inertia

Having explained how Orx stabilizes sleep and wake, and demonstrated that its loss produces sleep-wake fragmentation, in this final section we investigate how Orx affects the dynamics of state transitions. In our model, the timescale on which Orx dynamics occur, 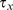, determines the timescale on which the MA receives an excitatory input upon awakening, and also the timescale on which this input is reduced following a transition to sleep. Here we show that this timescale selectively affects the sleep-to-wake transition, producing dynamics resembling sleep inertia, and discuss how the mechanism has a more general role in stabilizing arousal state changes, including naps during wake and awakenings during sleep.

The model predicts an asymmetry between the wake-to-sleep and sleep-to-wake transitions. In the original Phillips-Robinson model, the input to the MA was a constant, but in the current model, the input varies with Orx activity, which increases from a low value during a sleep-to-wake transition, and decreases from a high value during a wake-to-sleep transition. This change has a minimal effect on the wake-to-sleep transition, which occurs at high 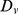 and 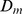 (cf. Fig. 3A), and is sharp, with the system attracting rapidly onto the sleep equilibrium. Once the system has begun to attract onto the sleep equilibrium, the threshold for a transition back to wake is very high and thus highly unlikely. By contrast, the sleep-to-wake transition is much more volatile because it occurs at low 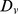 and 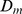 (cf. Fig. 3A) where the thresholds for state transitions are low. In addition, the circadian drive, 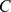, and hence Orx activity are both low in the morning, yielding low waking arousal levels, 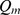, immediately following a normal morning awakening. For normal dynamics, the model therefore produces abrupt wake-to-sleep transitions but gradual and relatively volatile sleep-to-wake transitions (with the possibility of snoozing back to sleep). These qualitative dynamics resemble sleep inertia, a well-known phenomenon [87]–[90] that describes how “immediately after awakening from sleep, alertness is low” [91].

**Figure 6:**
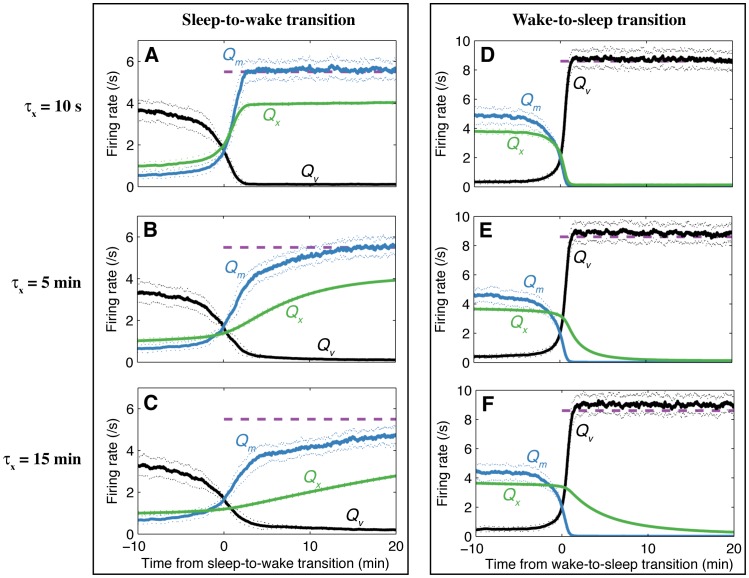
Dependence of sleep-to-wake and wake-to-sleep transitions on the timescale for Orx dynamics, 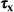. Time series for the firing rates of MA, 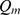 (blue), VLPO, 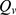 (black), and Orx, 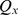 (green), are plotted for the sleep-to-wake (**A**–**C**) and wake-to-sleep (**D**–**F**) transitions for 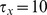 s (**A**, **B**), 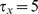 min (**B**, **E**), and 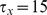 min (**C**, **F**), as a function of time relative to the change of state. The plots were produced by averaging 50 model runs relative to the time of the state transition; one standard deviation about the mean is shown dotted. The approximate steady state firing rate for 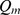 is annotated as a dashed purple line in **A**–**C**, and for 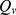 in **D**–**F**. The parameter 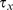 selectively tunes the duration of the sleep-to-wake transition but has minimal effect on the wake-to-sleep transition. This gradual wake transition can be linked to the clinical phenomenon of sleep inertia.

We find that the timescale for the sleep-to-wake transition depends on the timescale for Orx dynamics, 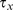. To demonstrate this, we plot time series for 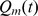, 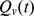, and 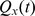 for normal sleep-to-wake and wake-to-sleep transitions in Fig. 6 for selected values of 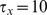 s, 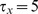 min, and 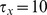 min. The time constants 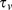 and 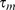 are maintained at their previous values of 10 s, for consistency with previous work (including the model’s response to external stimuli [39]). As shown in Figs. 6A–C, 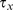 controls the timescale on which Orx activates during sleep-to-wake transitions, and hence that on which 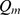, and waking arousal levels, increase to a steady level. This gradual sleep-to-wake transition stems from a longer timescale, 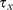, and constitutes a plausible mechanism for sleep inertia. Time series for wake-to-sleep transitions for the same three values of 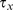 are plotted in Figs. 6D–F. Because normal wake-to-sleep transitions occur when 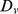 and 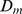 are high, the system attracts rapidly onto the sleep state, and the wake-to-sleep transition exhibits minimal dependence on 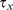. The parameter 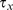 thus selectively tunes the timescale of sleep inertia in our model.

Depending on the study and the way sleep inertia is measured, its duration has been found to range from a few minutes to several hours, but in the absence of severe sleep deprivation, rarely exceeds 30 min [89]. For all simulations in this work, we set 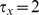 min, for which 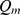 saturates over approximately 6 min following a normal morning awakening. The longer timescale for sleep inertia reported in some clinical studies [87], [88] may reflect the circadian input to Orx, which increases arousal levels, 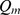, following an awakening on a longer timescale (to a maximum at the circadian peak in the mid-afternoon for a normal sleep-wake schedule, cf. Fig. 4A). Thus, while other models have used ad hoc processes to reproduce the dynamics of sleep inertia (e.g., the exponentially-saturating ‘inertia component’, 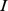 [91], [92]), here they emerge from modeling known physiological interactions.

As well as having a role in state transition dynamics, Orx controls the dynamics of all state transitions in the model, including naps during normal wake periods and awakenings during normal sleep periods. We first note that lasting changes of state could only occur in the original Phillips-Robinson model for the small range of drives, 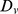, in the bistable region, due to a transient external stimulus, for example. By contrast, the new model is able to stabilize lasting changes of state when the system is not in the bistable region due to Orx, which activates to stabilize wake (increasing 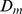), and deactivates to stabilize sleep (decreasing 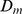) on a timescale 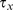. Thus, although changes in state can be produced by external drives acting on the relatively short timescale of 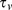 and 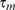, the system can remain in the new state after the external stimulus is removed if it persists on a timescale that is sufficiently long to change the activity of Orx (i.e., longer than 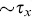). Intuitively, this behavior could correspond to relative difficulty returning to sleep after awakening in the night for more than a brief duration (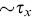), because this arousal persists for sufficiently long to activate Orx, stabilizing the wake state and preventing a rapid return back to sleep. Incorporating an excitatory input from Orx to MA in the model hence provides a more flexible framework for modeling state changes, with the time constant 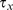 constituting a key timescale for both sleep inertia, and the stabilization of prolonged naps during wake and awakenings during sleep.

## Summary and Conclusion

In this work, a new model of sleep-wake physiology was developed that includes Orx. Using established physiological knowledge, the model addresses a key shortcoming in current understanding of narcolepsy by providing a clear physiological explanation of how arousal state instability stems from Orx loss. A physiologically plausible set of parameters is able to reproduce previously reported sleep-wake behavior, explain many features of the narcoleptic phenotype, and make new predictions. The main results are as follows:

- The new model produces realistic dynamics, including firing rates, relevant drives, and the temporal organization of sleep and wake periods.
- Fragmented sleep-wake time series characteristic of the narcoleptic phenotype are generated by simulating a reduction in orexin levels, yielding reduced daytime arousal with a constant daily total duration of sleep, without altering any other parameters or drives.
- The model predicts a shift of the sleep-wake schedule toward a morning chronotype with reduction in orexin levels, a prediction that may have relevance in understanding the increase in morningness and sleep-wake fragmentation with aging.
- While previous models have captured sleep inertia using ad hoc processes, an asymmetry between sleep-to-wake and wake-to-sleep transitions is predicted to result from adding Orx to the model, producing sleep inertia on the timescale of Orx dynamics, 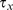. This timescale is shown to affect all state transitions, including naps during normal wake periods and awakenings during normal sleep periods.

Existing physiologically based models of sleep-wake dynamics have captured some elements of the role of Orx using alternative approaches. Unlike other models, our approach builds from a simplified model of the core physiology and does not attempt to include everything. This approach has the advantage of being able to model large networks of individual neurons as interacting populations, and producing easily-interpretable dynamics that reproduce many features of narcoleptic dynamics. In one model of mouse sleep-wake behavior by Diniz Behn *et al.* [30], Orx was modeled as a state-dependent modulation of the inhibition of the VLPO by wake-active neuronal populations using a saturating mathematical form that mimics Orx activation on a timescale of minutes or longer. This form ensures that Orx does not affect brief arousals, but only activates during extended wake periods (lasting longer than 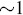 min) [30], [31]. Orx plays a qualitatively similar role in our model, but the dynamics result from directly modeling its interactions with other neuronal populations, including circadian input, which were not included in their model [30], [31]. Another physiologically based sleep model was proposed by Rempe *et al.* [33], that includes a similar set of neuronal populations and interactions as modeled here, but also included REM-off and REM-on populations, the eVLPO, and used a Morris-Lecar system to model each population as if it were an individual, representative cell. Their model includes Orx as a drive to monoaminergic nuclei that is, by construction, ‘switched on’ during wake (when it relays a purely circadian variation), and ‘switched off’ during sleep, whereas in our model Orx is included as a neuronal population with its own dynamics, more closely representing this aspect of the known physiology. The Rempe *et al.* [33] model successfully produced additional cycles between arousal states when removing the influence of Orx, but a relatively small number of features of the narcoleptic phenotype were reproduced.

Future modeling work could attempt to capture and account for the considerable inter-individual variation in narcoleptic symptoms [15] by relating changes in sleep-wake dynamics to changes in underlying model parameters (including 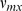, 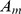, and 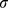, which all affect the rate of state transitions) using hypnograms recorded from narcoleptic dogs [93], mice [94], and humans [22], for example. Fitting the model to individual data may allow us to infer the degree of Orx loss, for example, with potential to recommend pharmacological or behavioral treatments to individuals. The model’s increased flexibility to simulate state changes also makes it well placed to investigate the statistics of wake and sleep bout durations, a subject that has received much attention [95]. This model could include pharmaceutical agents, as has been demonstrated for caffeine in the original Phillips-Robinson model [40]. For example, orexin receptor antagonists such as suvorexant [96] could be modeled straightforwardly, or modafinil could be modeled as increasing norepinephrinergic inhibition of the VLPO [97]. The model introduced here is thus flexible and well-placed to contribute to a unified understanding of a wide range of sleep-wake phenomena in terms of a simplified representation of the core underlying physiology.

## Supporting Information

File S1 **Additional modeling details.** This supplementary file contains additional mathematical detail of our the model, how its parameters have been constrained, a brief application to sleep deprivation, and a description of our heuristic for labeling ‘sleep’ and ‘wake’ periods.
(PDF)
