# Supplementary material for: A Physiologically Based Model of Orexinergic Stabilization of Sleep and Wake
Source: PLoS One. 2014 Mar 20;9(3):e91982. doi: 10.1371/journal.pone.0091982 (PMC3961294; doi:10.1371/journal.pone.0091982)
Supplement: File S1 — Additional modeling details. This supplementary file contains additional mathematical detail of our the model, how its parameters have been constrained, a brief application to sleep deprivation, and a description of our heuristic for labeling ‘sleep’ and ‘wake’ periods. (PDF) [file pone.0091982.s001.pdf]

Supporting File S1 for 'A physiologically based model of orexinergic stabilization of sleep and wake'

B. D. Fulcher[1*], A. J. K. Phillips[2], S. Postnova[1,3,4], P. A. Robinson[1,3,4,5]

1 School of Physics, The University of Sydney, Sydney, NSW 2006, Australia

2 Division of Sleep Medicine, Brigham & Women's Hospital, Harvard Medical School, 221 Longwood Ave., Suite 438, Boston, MA 02115, USA

3 Brain Dynamics Center, Sydney Medical School—Western, University of Sydney, Westmead, NSW 2145, Australia

4 Center for Integrated Research and Understanding of Sleep, 431 Glebe Pt. Rd., Glebe, NSW 2037, Australia

5 Cooperative Research Center for Alertness, Safety and Productivity, The University of Sydney, Australia

∗ **E-mail: ben.d.fulcher@gmail.com**

In this supplement we provide additional mathematical detail about the model, outline the process through which parameters are constrained, show that the model's predictions for sleep deprivation are consistent with previous results, and describe a simple heuristic used to label 'sleep' and 'wake' periods from the model output.

Nullclines and equilibriums

In this section we present equations that define the features of V_v–V_m plots, shown in Fig. 2 of the main text of the manuscript, and derived in previous work [1]. On timescales longer than τ_v and τ_m, D_v and D_m can be treated as control parameters of the fast model dynamics [1], which can be understood in terms of the nullclines $\dot{V}_v = 0$:

$$V_v = \nu_{vm} S(V_m) + D_v, \tag{1}$$

and $\dot{V}_m = 0$:

$$V_m = \nu_{mv} S(V_v) + D_m. \tag{2}$$

Note that in this work, since V_x contributes to D_m, this analysis also assumes that $\dot{V}_x \approx 0$ on the timescale of τ_m, τ_v, which will be a good approximation for $\tau_x \gg \tau_v, \tau_m$ (as is the case here). Equilibriums occur at $V_v = V_v^*$ and $V_m = V_m^*$, at the intersection of these two nullclines, and can be written implicitly in terms of V_v^* as

$$-V_v^* + \nu_{vm} S\left[\nu_{mv} S(V_v^*) + D_m\right] + D_v = 0, \tag{3}$$

or in terms of V_m^* as

$$-V_m^* + \nu_{mv} S\left[\nu_{vm} S(V_m^*) + D_v\right] + D_m = 0. \tag{4}$$

Saddle-node bifurcations occur in the model at different combinations of D_v and D_m [1, 2]. To compute the bifurcation boundaries in D_v–D_m space, plotted throughout this work, we first solved for V_v for a

given $D_m = D_m^{\mathrm{bif}}$, using

$$-1/(\nu_{vm}\nu_{mv}) + S'[\nu_{mv}S(V_v) + D_m^{\mathrm{bif}}]S'(V_v) = 0, \tag{5}$$

where $S'(x) = dS(x)/dx$. Equation (5) has solutions $V_v = V_v^{\mathrm{bif}}$, which allows us to then evaluate the corresponding drives at which bifurcations occur, D_v^{bif}, using

$$D_v^{\mathrm{bif}} = V_v^{\mathrm{bif}} - \nu_{vm}S\left[\nu_{mv}S(V_v^{\mathrm{bif}}) + D_m^{\mathrm{bif}}\right]. \tag{6}$$

The unstable manifold, W^+, of a saddle point equilibrium, (V_v^*, V_m^*), forms a separatrix between sleep and wake basins in V_v–V_m space. It is calculated numerically here by incrementally moving in the direction the negative of the vector field, $(-\dot{V}_v, -\dot{V}_m)$, after small positive and negative perturbations from the unstable equilibrium with the gradient $m_+ = dV_m/dV_v = \sqrt{\nu_{vm}S'(V_v^*)/\nu_{mv}S'(V_m^*)}$, as derived using the Stable Manifold Theorem in previous work [1]. That is, $(-\dot{V}_v, -\dot{V}_m)$ is followed from initial points $(V_v^* + \epsilon, V_m^* + m_+\epsilon)$ and $(V_v^* - \epsilon, V_m^* - m_+\epsilon)$, where ϵ is the small perturbation. A value $\epsilon = 0.01\,\mathrm{mV}$ is used here.

Parameter Constraints

The process through which the new structure and parameters of this model are constrained is explained in this section. Compared to the original Phillips-Robinson model, the current model includes several new parameters, as well as some adjustments of existing parameters, see Table 1 in the main text for a list of all parameter values used here. Most parameters are kept to existing values and we maintain as much compatibility as possible with previous work, ensuring that key successes of the previous model, including normal dynamics (characterized in the main text) and sleep deprivation (later in this supplement), remain consistent. Parameters that are new or have been altered from the original Phillips-Robinson model can be grouped as follows: (i) *Orx dynamics*: Orx dynamics occur on a timescale τ_x, Orx is inhibited by the VLPO, ν_{xv}, Orx receives an excitatory circadian projection, ν_{xc}, provides an excitatory input to the MA, ν_{mx}, and receives a constant input, A_x, (ii) *Sleep homeostatic production form*: the strength of production, μ_h, and its saturation scale, η_h (used previously in [3]), (iii) *Constant drives*: to the MA, A_m, and VLPO, A_v are altered, (iv) *Circadian input to the VLPO*: ν_{vc} is reduced, and (v) *Noise processes*: added to the VLPO and MA with standard deviation σ.

When simulating narcoleptic dynamics, we set $\nu_{mx} = 0$, so that the parameters controlling Orx dynamics have no effect on the sleep-wake switch. This allows a reduced number of parameters: A_m, ν_{vc}, and A_v, to be constrained. The parameters $A_m(= 0.52\,\mathrm{mV})$ and $A_v(= -8.5\,\mathrm{mV})$, are set so that narcoleptic dynamics occur where thresholds for sleep-wake transitions are low, and could be modified to match clinical data for narcoleptics in future work. The 60% reduction in A_m compared to previous work implies that approximately 60% of A_m (the time-averaged drives to the MA modeled in previous work) can be attributed to Orx. In this model, the circadian input to the sleep-wake switch is now split between Orx and the VLPO, with a dominant pathway to Orx. The magnitude of the parameter ν_{vc} is correspondingly lower than in previous work (i.e., $\nu_{vc} = -2.9\,\mathrm{mV\,s} \rightarrow \nu_{vc} = -0.29\,\mathrm{mV\,s}$), motivated by

the observation that circadian rhythmicity is strong in patients with VLPO legions [4]. The circadian phase dependence on sleep and wake for narcoleptics depends on A_m and A_v, and most importantly the oscillation magnitude ν_{vc}.

Having fitted these parameters to reproduce key features of the narcoleptic phenotype, the remaining parameters are constrained by fitting to normal dynamics. Dynamical parameters for Orx are set to reproduce normal sleep-wake dynamics: $\tau_x(= 2\,\mathrm{min})$ is set to reproduce an approximate timescale for sleep inertia (as explained in the main text), $\nu_{xc}(= 1.0\,\mathrm{mV\,s})$ is set to provide an appropriate amount of circadian variation in waking arousal levels, $\nu_{mx}(= 0.3\,\mathrm{mV\,s})$ is set to relay an appropriate circadian variation to the MA during waking, and $A_x(= 1.0\,\mathrm{mV})$ is set to a value that allowed the system to wake up in the early morning when the circadian drive is low. The parameters, μ_h and η_h are constrained by maintaining an approximately equal amount of time spent awake across the range of ν_{mx}, that is, for both normals (that have $Q_m \approx 6\,\mathrm{s}^{-1}$ during wake) and narcoleptics (that have $Q_m \approx 2.4\,\mathrm{s}^{-1}$ during wake). The noise standard deviation, $\sigma = 1\,\mathrm{mV}$, is chosen to produce a realistic rate of arousal-state fragmentation in the narcoleptic phenotype.

Note that although many of the model parameters are set to values that produce sensible dynamics, they could be constrained more thoroughly, and also fitted to the sleep patterns of individuals, using clinical data. For example, the model could be fitted to state transition statistics and sleep-wake timings obtained from narcoleptic patients, as well as potentially constraining model parameters directly, using physiological data (e.g., of neuronal population firing rates in sleep and wake). We emphasize that our aim in this work is not to perform a thorough fitting of the model to real data, but to present a physiologically-plausible set of parameters that produces dynamics consistent with the known sleep-wake behavior, including how changes in Orx affect the dynamics.

The notation used in this work differs somewhat from previous work on the original Phillips-Robinson model [5]. In previous work, the only time-varying drive to the system was an input to the VLPO; in this work we have introduced a consistent labeling across the model equations for MA (m), VLPO (v), and Orx (x). For example, we label D (original model) as D_v (current model), D_0 (original model) as A_v (current model), and A (original model) as A_m (current model).

Sleep deprivation

Because the model structure is different to that of previous work—with the addition of the Orx neuronal population and the dominant circadian input now acting through this population—we want to ensure that the behavior of the new model is consistent with previous results. Although it is beyond the scope of the current work to apply the new model to the full set of previously modeled phenomena, we have tested for consistency with both normal sleep-wake behavior and sleep deprivation. In this section, we characterize a simulated *wake effort* time series for sleep deprivation using the new model with Orx. The wake effort is defined as the minimal additional drive required to be applied to MA to maintain the system at a stable wake equilibrium [3]. As shown in Fig. 1, despite the presence of Orx in the model and that the dominant C drive is now afferent to MA rather than VLPO, the model produces the same pattern

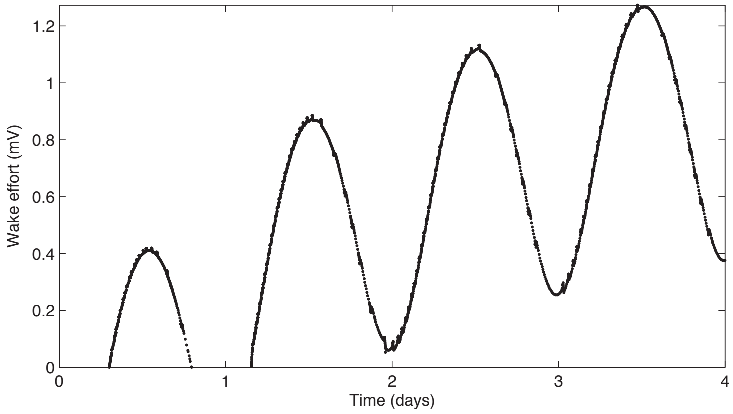

Figure S1. The new model reproduces the pattern of 'wake effort' across a period of extended sleep deprivation found in previous work [3]. The system is maintained in a waking state during normal sleep periods by applying an additional wake effort drive to the MA. The resulting dynamics are maintained despite changes in the model's structure, including a dominant circadian input to Orx to MA rather than a sole input to the VLPO, as in the original Phillips-Robinson model. The roughness of the curves is an artifact of the numerical implementation used here.

for the wake effort, with an oscillation superimposed on a increasing trend, but returning to baseline after the first night of total sleep deprivation [3]. The simulation is performed for nominal parameters without added noise by maintaining the system in the bistable region (and hence awake) whenever the system reaches the sleep bifurcation boundary. Although the Phillips-Robinson model only had a single time-varying drive, D_v, sleep deprivation is simulated here in the same way: driving the system to wake using the minimal input to the MA [3].

Heuristic for labeling sleep and wake states

In this work, 'sleep' and 'wake' states sometimes need to be labeled from the output of our model, which produces time series for V_v, V_m, and V_x. For this task, we use a simple heuristic that divides the model output into non-overlapping 20 s windows and performs a preliminary classification of each window as 'wake' if $V_m > V_v$ more than half of the time, and 'sleep' otherwise. We then discounted bouts lasting less than 60 s as transient (indicative of the model not yet settling on a stable state), ignoring such brief interludes by labeling them as if they did not occur. The overall labeling produced by this procedure is not very sensitive to changes in these two parameters (i.e., the window length and minimum bout duration) across a range of reasonable choices.

Acknowledgments

This work was supported by the Australian Research Council (ARC), National Health and Medical Research Council (NHMRC), Westmead Millennium Institute, Brain Resource Ltd, and by the National Space Biomedical Research Institute through NASA NCC 9-58.

References

1. B. D. Fulcher, A. J. K. Phillips, and P. A. Robinson. Modeling the impact of impulsive stimuli on sleep-wake dynamics. *Phys. Rev. E* **78**, 051920 (2008).

2. S. H. Strogatz. *Nonlinear Dynamics and Chaos: With Applications to Physics, Biology, Chemistry, and Engineering.* Westview Press (1994).

3. B. D. Fulcher, A. J. K. Phillips, and P. A. Robinson. Quantitative physiologically based modeling of subjective fatigue during sleep deprivation. *J. Theor. Biol.* **264**, 407 (2010).

4. J. Lu, M. Greco, P. Shiromani, and C. B. Saper. Effect of lesions of the ventrolateral preoptic nucleus on NREM and REM sleep. *J. Neurosci.* **20**, 3830 (2000).

5. A. J. K. Phillips and P. A. Robinson. A quantitative model of sleep-wake dynamics based on the physiology of the brainstem ascending arousal system. *J. Biol. Rhythms* **22**, 167 (2007).
